# Multiperspective quantitative tumor–stroma ratio reveals histological areas associated with poor outcomes in oral squamous cell carcinoma

**DOI:** 10.1002/cam4.5909

**Published:** 2023-05-15

**Authors:** Shuai Wang, Qian Si, Yan Wu, Yawei Sun, Weixian Zhang, Xiaofeng Huang, Tao Zeng, Sheng Chen, Xihu Yang, Yanhong Ni, Qingang Hu

**Affiliations:** ^1^ Central Laboratory of Stomatology Nanjing Stomatological Hospital, Medical School of Nanjing University Nanjing Jiangsu China; ^2^ Department of Oral and Maxillofacial Surgery Nanjing Stomatological Hospital, Medical School of Nanjing University Nanjing Jiangsu China; ^3^ Department of Oral Pathology Nanjing Stomatological Hospital, Medical School of Nanjing University Nanjing Jiangsu China; ^4^ State Key Lab of Pharmaceutical Biotechnology, College of Life Sciences Nanjing University Nanjing Jiangsu China; ^5^ Department of Oral and Maxillofacial Surgery Affiliated Hospital of Jiangsu University Zhenjiang Jiangsu China

**Keywords:** inner tumor, invasive tumor front, oral squamous cell carcinoma, tumor‐stroma ratio

## Abstract

**Aims:**

Different regions of oral squamous cell carcinoma (OSCC) have particular histopathological characteristics, and the individual histological characteristics of the tumors are poorly understood. Therefore, calculating the proportion of tumor cells in different regions that allow assessment of the prognostic outcomes for OSCC patients would be of great clinical significance.

**Methods and Results:**

We established an open‐source software‐based analytic pipeline that defines the inner tumor and invasive tumor front (ITF) in pancytokeratin‐stained whole slide images (WSIs) and quantifies the tumor‐stroma ratio (TSR) within the two regions. We applied this method to 114 patients with OSCC and predicted patient prognosis by the TSR. The proportion of tumor area in the inner tumor was generally higher than that in the ITF (*p* < 0.0001). TSR was an independent prognostic factor for overall survival (OS) (*p* = 0.016), disease‐free survival (DFS) (*p* = 0.026), and relapse‐free survival (RFS) (*p* = 0.037) in inner tumor, and TSR was an independent prognostic factor for OS (*p* = 0.00052), DFS (*p* = 0.035), and metastasis‐free survival (MFS) (*p* = 0.038) in the ITF. Tumor‐low status was associated with poorer prognosis. There was a significant correlation between the TSR and perineural invasion (PNI) in the inner tumor (*p* = 0.009).

**Conclusions:**

The histopathological characteristics of different regions of OSCC may be used to develop the potential prognostic markers. The TSR of the inner tumor is more targeted in predicting prognosis and accurately assesses the risk of PNI+.

## Graphical abstract

Different regions of oral squamous cell carcinoma (OSCC) have particular histopathological and molecular characteristics. Histopathological‐based models may be used to stratify patients into low‐ and high‐risk classes. TSRs of the inner tumor and invasive tumor front can predict the prognosis of OSCC patients.

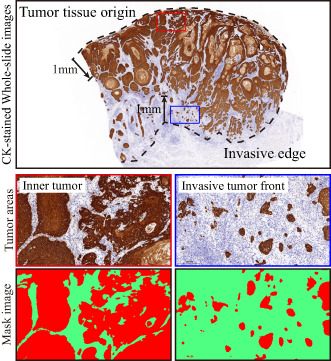

## 1 INTRODUCTION

Oral squamous cell carcinoma (OSCC) often leads to oral dysfunction and deformed appearance in patients, greatly reducing the quality of life of these individuals and even causing death. At present, the prognostic stratification of OSCC patients mainly depends on the TNM staging system. The process of tumor occurrence and development is multifactorial, and one of the important factors is the interaction between the tumor and the microenvironment,^1^ which is not clearly reflected in the TNM staging system.

The tumor stroma plays an important role in the occurrence, progression, metastasis, and drug resistance of cancer and is closely related to the prognosis of patients,^2^ which also shows that it has high prognostic value. The ratio between tumor cells and tumor‐associated stroma in tumor tissue is defined as the tumor–stroma ratio (TSR). Several studies have shown that the TSR is related to the prognosis of OSCC, but except for one study using semiautomatic analysis,^3^ the TSR of other studies was visually evaluated by pathologists under a microscope on HE‐stained slides.^4^, ^5^, ^6^, ^7^, ^8^ Standardized and repeatable prognostic factors are an important part of clinical cancer research and one of the pillars of personalized medical care. Therefore, through objective and standardized methods, a TSR quantization method suitable for OSCC can be developed with the best repeatability and application scope.

OSCC has obvious morphological heterogeneity, which is related to its characteristics of invasion and stroma composition polymorphism. It has been well reported that the invasive tumor front (ITF) and inner tumor are different in histomorphology and molecular characteristics.^9^, ^10^ Compared with the inner tumor, the differentiation degree of tumor cells in the ITF is lower, the dissociation and motor ability of cells are stronger, and the EMT characteristics are more obvious.^11^ Therefore, more studies report that the ITF is the key region for the dynamic progression of malignant tumors.^12^ However, as a comparative factor, the role of histomorphology of inner tumor in judging the malignant degree of OSCC cannot be ignored.^13^, ^14^ In addition, both TSR quantification based on whole‐slide images (WSIs) and TSR quantification based on ITF alone have shown good prognostic prediction,^3^, ^14^ which also indicates that the inner tumor region may have the ability to predict prognosis. This ability may play an important role when the ITF cannot be determined in tissue slides.

Therefore, we aimed to quantify the TSR of the inner tumor and ITF in CK‐stained OSCC tissue sections and evaluate the prognostic prediction power of the TSR for OSCC survival and its relationship with clinicopathological factors.

## 2 MATERIALS AND METHODS

### 2.1 Study population

All study cases were composed of randomly selected OSCC patients. All patients received radical resection in the Department of Oral and Maxillofacial Surgery of Nanjing Stomatological Hospital from 2015 to 2019. Clinical and pathological data were obtained from the archives of the tissue sample database of Nanjing Stomatological Hospital. The inclusion criteria were as follows: (1) primary OSCC without any treatment; (2) no human papillomavirus (HPV) infection (evaluated by HPV16‐specific fluorescence in situ hybridization and p16Ink4a‐specific immunohistochemistry); (3)patients were followed up for more than 3 years. The exclusion criteria were as follows:(1) patients who died within 30 days after the operation; (2)tissue‐section tumor thickness that was less than 2 mm; and (3)patients with a lack of clinical information (regarding mortality or time). All patients were staged and classified according to the eighth edition of the AJCC staging system.^15^

### 2.2 Tissue slide preparation and scanning

Hematoxylin and eosin (HE)‐stained sections (2 μm) were obtained from formalin‐fixed paraffin‐embedded blocks of primary tumor specimens. The slices were selected from the most aggressive part of OSCC (i.e., the same slides routinely used to evaluate T status). In general, the pathology department used the continuous slides of this section to prepare various immunohistochemical sections (including CK 5/6). Therefore, if there was already a histochemically stained section corresponding to the selected HE section, we used this section. If not, then immunohistochemical staining of cytokeratin AE1/AE3 was prepared according to our routine protocol, which was described in a previous report.^16^ After all staining was completed, digital HE and CK‐stained sections of primary OSCC were reviewed by CaseViewer 2.4 for Windows (3DHISTECH Ltd.), a digital application for evaluating microscopic images. HE‐ and CK‐stained slides were scanned into high‐resolution (0.14 μm/pixel) digital WSIs at 40× magnification using a 3DHistech Pannoramic MIDI II scanner (3DHISTECH Ltd.).

### 2.3 Construction of image fields for evaluating the TSR

**FIGURE 1:**
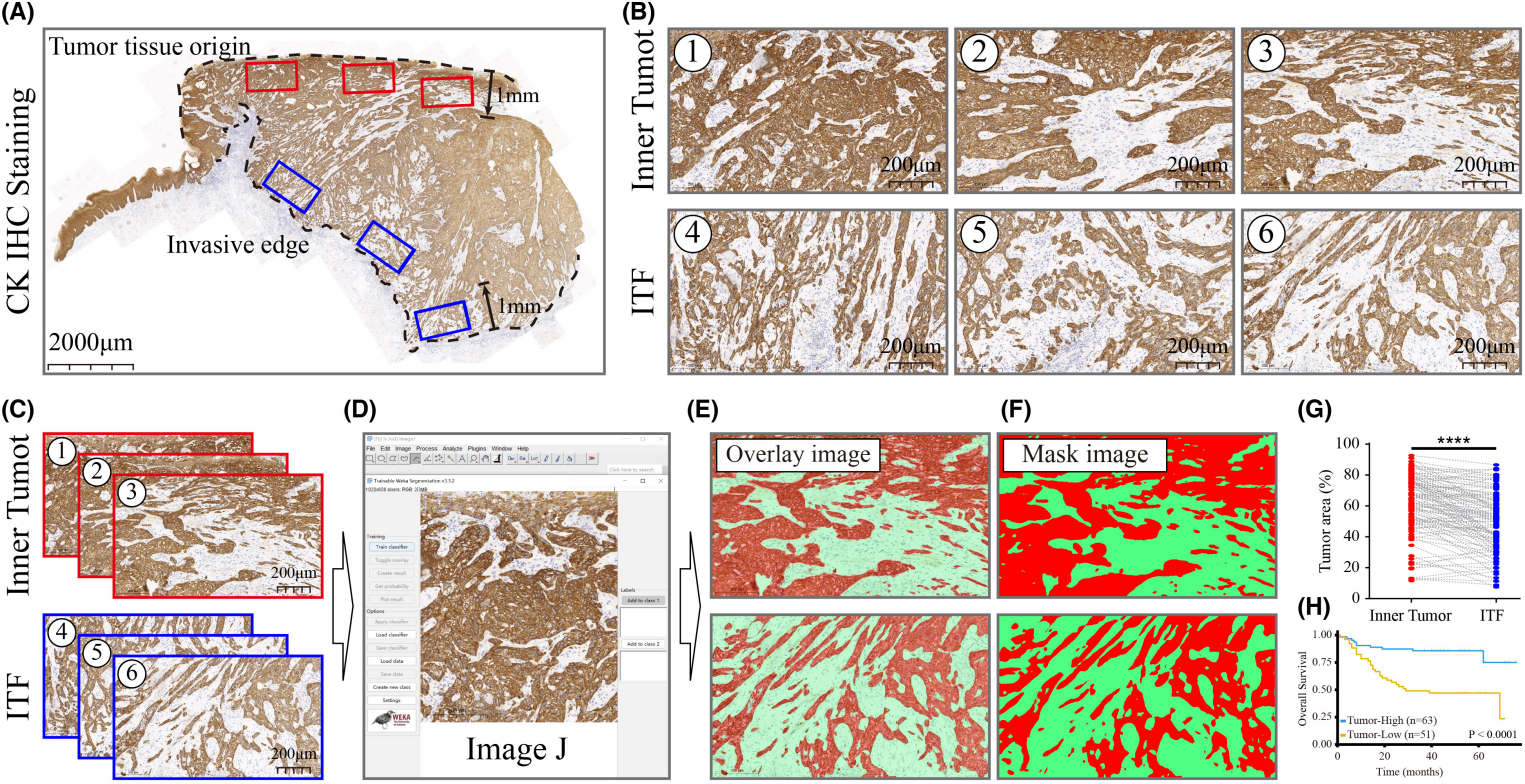
Slide analysis workflow. (A) Three inner tumor image fields and three ITF image fields were selected in the WSI by CaseViewer software. The upper and lower dotted lines represent the tumor tissue origin and the invasive edge, respectively, and the 1 mm zone extending from the upper and lower dotted lines to the center of the tumor is the inner tumor and ITF. (B) Six image fields were selected by pathologists who were blinded to the clinical information: 1–3 are representative image fields of the inner tumor, and 4–6 are representative image fields of the ITF. (C–F) The tissue of the pathological image was segmented, recognized and classified by the Trainable Weka Segmentation (TWS) plug‐in of ImageJ to determine the tumor area, and finally, a binary mask image (F) was obtained, according to which the area ratio of tumor cells was calculated. (G) Paired *t*‐test results of the proportion of tumor area of the inner tumor and ITF. (H) The prognosis of OSCC patients was predicted according to the calculated proportion of tumor area.

We first defined two tumor histological regions in CK‐stained WSIs.^1^ Inner tumor: 1 mm zone extending from the origin of the epithelial tumor tissue to the deep part of the tumor.^2^ ITF: 1 mm zone extending from the invasive edge to the inside of the tumor.^9^, ^17^ The tumor side (relative to the normal side) was enclosed in the tumor area, and the normal tissue side was in contact with the surrounding normal tissue, which is different from the traditional ITF concept^18^ (Figure 1A). Based on the literature and our experience in routine pathological diagnosis, we chose an area equivalent to the 20× objective selection of most light microscopes (length: 1.15 mm, width: 0.68 mm, area: 0.78 mm^2^).^14^ In practice, it is difficult for pathologists to quickly and precisely locate invasion areas with the lowest proportion of tumor cell distribution on tissue slides. To more realistically simulate the process of clinical selection of the image views, we selected invasive areas with the lowest possible percentage of tumor cell distribution by a single‐blind (independent of the purpose of this study) pathologist in CaseViewer software. Tumor cells were present at all borders of the selected image field. Three image fields were selected for the inner tumor and ITF (Figure 1A,B), and as much as possible, the distribution density of tumor cells in the three image fields was the lowest in the inner tumor and ITF. To ensure that the image field with the highest proportion of stroma could be selected, we allowed an overlap between image fields. In fact, the incidence of this situation is not high, about 5.3% (6/114).

### 2.4 Evaluation of tumor distribution density

After selecting the image field, the tissue image of the corresponding area was extracted using slide‐reading software (Figure 1C). In a further step, the binarization transformation of the tissue image was implemented through the TWS plug‐in^19^, ^20^, ^21^ of ImageJ software (Figure 1D–F). In short, the area of the tumor cells was measured on brown‐stained paraffin slides using a trainable segmentation method based on the WEKA TWS plug‐in. Two subcategories were created: tumor cells and tumor stroma (Figure 1E). For regions that were not classified accurately, we manually added the labels needed by the classifier until the classification was accurate. With the obtained mask images, the data of the tissue image and tumor area could be easily calculated (Figure 1F), and the proportion of tumor area (tumor area/image field area) could be obtained.

### 2.5 TSR definition

In this study, the TSR was defined as the tumor cell area/corresponding image field area × 100%. There were two types of TSR in this study: (1) TSR of the inner tumor and (2) TSR of the ITF. Because the two types of TSR have three image fields, each can calculate three TSR values. For statistical analysis and to simulate possible clinical bias in the results, we averaged the three TSR values as the TSR values for the final analysis. Subsequently, the patients were divided into a tumor‐high group and a tumor‐low group; the former was defined as a high proportion of tumor area and a low proportion of stroma (TSR ≥50%), while the latter was defined as a low proportion of tumor area and a high proportion of stroma (TSR <50%).

### 2.6 Statistical analysis

The baseline data table, KM curve, Cox proportional risk model, and binary logistic regression analysis of the study cohort were all performed in R (R version 4.2.0) using the following R software packages: Tableone, survival, and blorr. The correlation between TSR and clinicopathological variables was completed in GraphPad Prism 8.0 (GraphPad Software Inc.), and Spearman grade correlation analysis was used. A paired *t*‐test was used to analyze the difference in TSR expression between the inner tumor and ITF. To study the role of clinicopathological parameters in predicting PNI, a logistic regression model was used for univariate and multivariate analyses. The odds ratio (OR) and its 95% confidence interval (95% CI) were calculated. The primary endpoint was to detect any significant difference in survival between cases in the tumor‐high group (high TSR) and the tumor‐low group (low TSR). All clinical parameters and TSRs were used as classification variables to evaluate the correlation between the TSR and clinicopathological variables. Univariate Cox regression analysis was used to estimate the correlation between variables and survival outcomes (overall survival [OS], disease‐free survival [DFS], metastasis‐free survival [MFS], and relapse‐free survival [RFS]). In addition, a multivariate Cox proportional risk model was established to evaluate the correlation between statistically significant predictive variables in univariate analysis and their influence on prognosis. All statistical tests were two‐sided, and statistical significance was defined as *p* < 0.05.

## 3 RESULTS

### 3.1 Demographic and clinicopathological variables

Detailed clinicopathological features of the patients are listed in Table 1. In this study, a total of 114 OSCC patients who received radical resection in the Department of Oral and Maxillofacial Surgery of Nanjing Stomatological Hospital from 2015 to 2019 were included. The mean follow‐up time was 52.35 ± 20.04 months (range, 1–76 months). Among them, 38 (33.33%) patients died of OSCC, 39 (34.21%) patients had recurrence or metastasis, 15 (13.16%) patients had recurrence, and 25 (21.93%) patients had metastasis. Paired *t*‐test results showed that the TSR of inner tumor was significantly higher than the ITF (*p* < 0.0001) (Figure 1G). The correlation analysis of the TSR (as a categorical variable) with other clinicopathological variables (sex, age, clinical grade, tumor differentiation, smoking, pattern of invasion [POI], worst POI [WPOI], DOI, and PNI) showed that the POI and WPOI of patients in the tumor‐low group were higher than those of patients in the tumor‐high group, and the DOI and PNI also showed the same trend.

**TABLE 1:** Distribution of demographic and clinicopathological characteristics of OSCC patients in the inner tumor and ITF.

Characteristics	*n* (%)	Inner tumor		p*	ITF		p*
		Tumor‐high (%)	Tumor‐low (%)		Tumor‐high (%)	Tumor‐low (%)	
Sex							
Male	69 (60.5)	51 (60.0)	18 (62.1)	1	40 (63.5)	29 (56.9)	0.598
Female	45 (39.5)	34 (40.0)	11 (37.9)		23 (36.5)	22 (43.1)	
Age							
<60	44 (38.6)	35 (41.2)	9 (31.0)	0.455	26 (41.3)	18 (35.3)	0.647
≥60	70 (61.4)	50 (58.8)	20 (69.0)		37 (58.7)	33 (64.7)	
Stage							
I	8 (7.0)	7 (8.2)	1 (3.4)	0.724	5 (7.9)	3 (5.9)	0.926
II	24 (21.1)	17 (20.0)	7 (24.1)		13 (20.6)	11 (21.6)	
III	20 (17.5)	16 (18.8)	4 (13.8)		12 (19.0)	8 (15.7)	
IV	62 (54.4)	45 (52.9)	17 (58.6)		33 (52.4)	29 (56.9)	
Differentiation							
Well	85 (74.6)	67 (78.8)	18 (62.1)	0.123	47 (74.6)	38 (74.5)	1
Moderate‐poor	29 (25.4)	18 (21.2)	11 (37.9)		16 (25.4)	13 (25.5)	
Smoking							
No	68 (59.6)	50 (58.8)	18 (62.1)	0.93	37 (58.7)	31 (60.8)	0.976
Yes	46 (40.4)	35 (41.2)	11 (37.9)		26 (41.3)	20 (39.2)	
POI							
1–3	64 (56.1)	53 (62.4)	11 (37.9)	***0.038***	44 (69.8)	20 (39.2)	***0.002***
4–5	50 (43.9)	32 (37.6)	18 (62.1)		19 (30.2)	31 (60.8)	
WPOI							
1–3	30 (26.3)	24 (28.2)	6 (20.7)	0.581	22 (34.9)	8 (15.7)	***0.035***
4–5	84 (73.7)	61 (71.8)	23 (79.3)		41 (65.1)	43 (84.3)	
DOI							
<5 mm	34 (29.8)	28 (32.9)	6 (20.7)	0.312	22 (34.9)	12 (23.5)	0.264
≥5 mm	80 (70.2)	57 (67.1)	23 (79.3)		41 (65.1)	39 (76.5)	
PNI							
No	80 (70.2)	68 (80.0)	12 (41.4)	***<0.001***	53 (84.1)	27 (52.9)	***0.001***
Yes	34 (29.8)	17 (20.0)	17 (58.6)		10 (15.9)	24 (47.1)	

Abbreviations: DOI, depth of invasion; ITF, invasive tumor front; PNI, perineural Invasion; POI: pattern of invasion; WPOI, worst POI. 1

### 3.2 Evaluation of the prognostic value of the TSR

**FIGURE 2:**
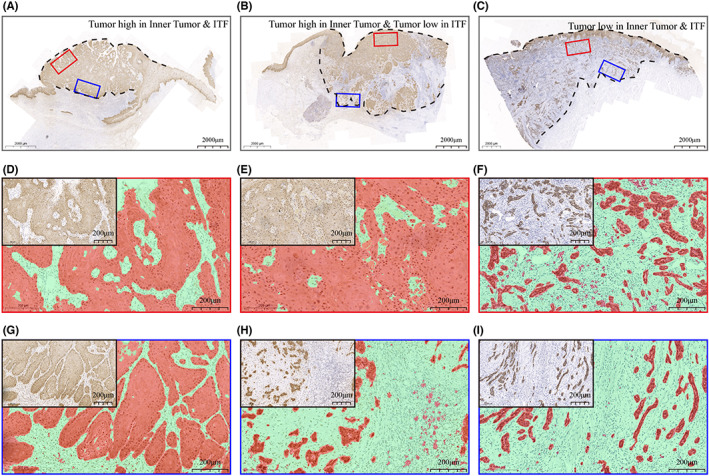
Three conditions of the proportion of tumor area in OSCC. (A) WSI with a high TSR of the inner tumor and ITF. (B) WSI with a high TSR in the inner tumor and a low TSR in the ITF. (C) WSI with a low TSR of the inner tumor and ITF. (D–F) Enlarged images of the inner tumor image fields corresponding to (A)–(C) and the overlay images after ImageJ segmentation. (G–I) Enlarged images of the ITF image fields corresponding to (A)–(C) and the overlay images after ImageJ segmentation.

The TSR differed significantly in the inner tumor and ITF (Figure 1G), and this difference was not consistent (Figure 2). We categorized it into three general situations: (1) a high TSR for both the inner tumor and the ITF (Figure 2A,D,G); (2) a high TSR for the inner tumor and a low TSR for the ITF (Figure 2B,E,H); and (3) a low TSR for both the inner tumor and the ITF (Figure 2C,F,I). Therefore, in the survival analysis, we analyzed the prognostic value of the TSR of the inner tumor and ITF separately.

**FIGURE 3:**
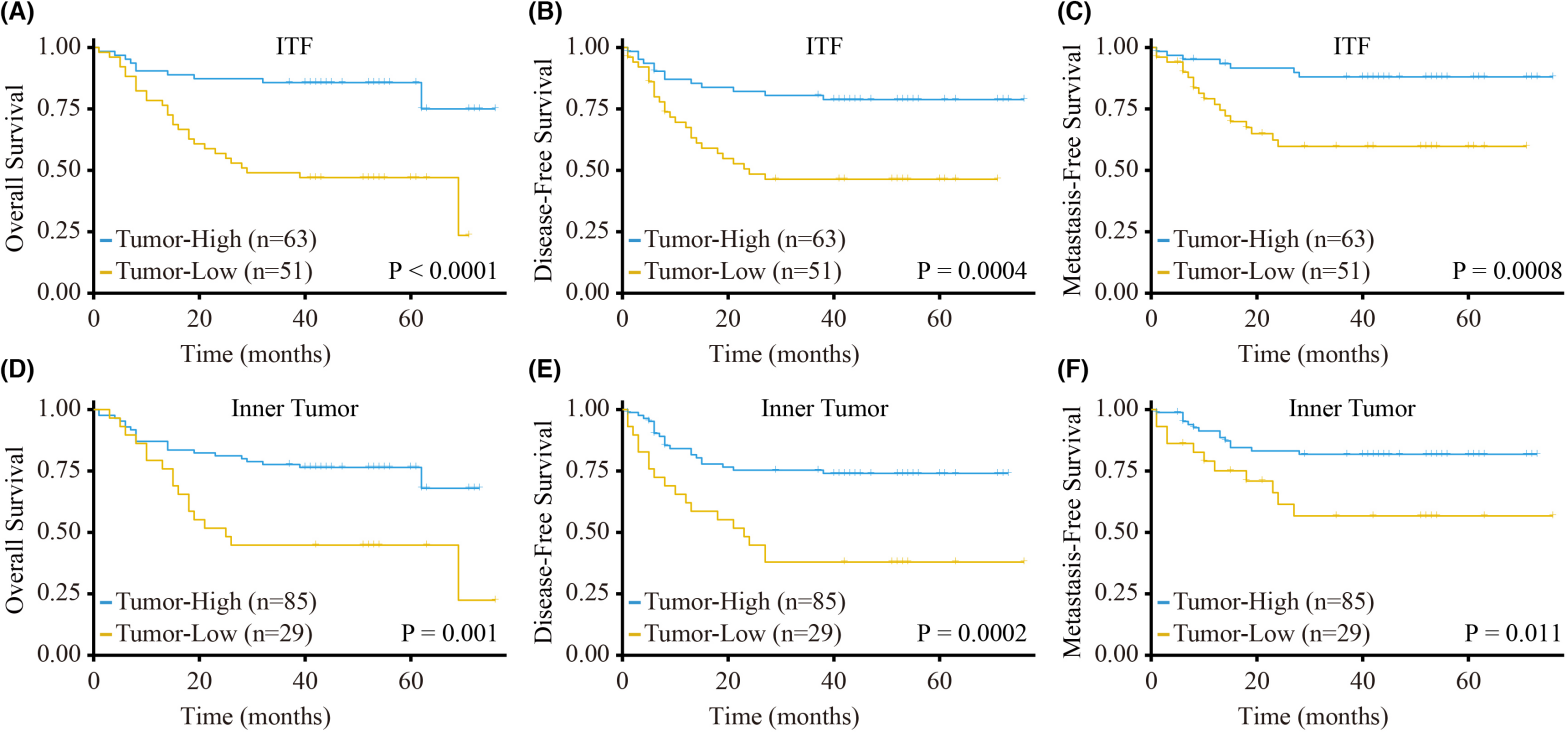
Differential prognoses of the TSR in the inner tumor and ITF. (A–C) K–M curves of the TSR of the ITF; OS (A), DFS (B) and MFS (C) were significantly lower in the tumor‐low group (*n* = 51) than in the tumor‐high group (*n* = 63). (D–F) K–M curves of the TSR of the inner tumor; OS (D), DFS (E) and MFS (F) were significantly lower in the tumor‐low group (*n* = 29) than in the tumor‐high group (*n* = 85).

For the ITF, the results of the Cox proportional hazards model showed that OS was significantly lower in patients in the tumor‐low group (low TSR) than in OSCC patients in the tumor‐high group (high TSR), with a hazard ratio (HR) of 4.34, 95% CI of 2.10–8.97 (*p* < 0.0001), DFS (HR = 3.13, 95% CI 1.60–6.1; *p* = 0.00082), and MFS (HR = 4.01, 95% CI 1.67–9.63; *p* = 0.0019) were poor (Figure 3A–C, Tables 2, Table S1, Figure S1A). In multivariate analysis, the TSR was an independent prognostic factor for OS (HR = 3.96, 95% CI 1.82–8.60; *p* = 0.00052), DFS (HR = 2.21, 95% CI 1.06–4.64; *p* = 0.035), and MFS (HR = 2.81, 95% CI 1.06–7.46; *p* = 0.038) after adjusting for sex, age, clinical stage, differentiation, smoking, POI, WPOI, DOI, and PNI (Tables 2, Table S1).

**TABLE 2:** Univariate and multivariate analyses including sex, age, clinical stage, differentiation, smoking, POI, WPOI, DOI, PNI and OS and DFS of two types of TSR.

	Univariate analysis (inner Tumor & ITF)							Multivariate analysis (ITF)							Multivariate analysis (inner tumor)						
	Overall survival				Disease‐free survival			Overall survival				Disease‐free survival			Overall survival				Disease‐free survival		
	HR	95% CI	p*		HR	95% CI	p*	HR	95% CI	p*		HR	95% CI	p*	HR	95% CI	p*		HR	95% CI	p*
Sex																					
Male	1				1																
Female	0.93	0.48–1.79	0.82		0.83	0.43–1.59	0.57														
Age																					
<60	1				1																
≥60	1.64	0.81–3.32	0.17		1.34	0.69–2.61	0.39														
Stage																					
I	1				1																
II	2.12	0.25–18.33	0.49		1.92	0.22–16.42	0.55														
III	2.87	0.34–23.97	0.33		3.81	0.48–30.45	0.21														
IV	4.44	0.6–32.88	0.14		4.14	0.56–30.56	0.16														
Differentiation																					
Well	1				1																
Moderate‐poor	1.15	0.56–2.38	0.70		0.9	0.43–1.9	0.79														
Smoking																					
No	1				1																
Yes	1.02	0.53–1.98	0.94		1.15	0.61–2.17	0.66														
POI																					
1–3	1				1			1				1			1				1		
4–5	2.34	1.22–4.5	***0.011***		2.58	1.35–4.92	***0.0041***	1.67	0.82–3.40	0.16		1.65	0.81–3.34	0.17	1.95	0.96–3.97	0.066		1.9	0.95–3.8	0.072
WPOI																					
1–3	1				1																
4–5	1.71	0.75–3.9	0.20		1.51	0.69–3.29	0.3														
DOI																					
<5 mm	1				1			1				1			1				1		
≥5 mm	3.37	1.31–8.65	***0.011***		2.95	1.23–7.04	***0.015***	2.98	1.13–7.87	***0.028***		2.2	0.89–5.42	0.087	2.93	1.09–7.93	***0.034***		2.1	0.83–5.28	0.11
	HR	95% CI	p*		HR	95% CI	p*	HR	95% CI	p*		HR	95% CI	p*	HR	95% CI	p*		HR	95% CI	p*
PNI																					
No	1				1			1				1			1				1		
Yes	2.03	1.06–3.86	***0.032***		2.87	1.53–5.38	***0.001***	0.72	0.34–1.53	0.4		1.37	0.65–2.87	0.41	0.79	0.35–1.78	0.56		1.28	0.58–2.83	0.54
Inner tumor																					
Tumor‐High	1				1										1				1		
Tumor‐Low	2.78	1.46–5.28	***0.0018***		3.08	1.64–5.79	***0.00047***								2.46	1.18–5.13	***0.016***		2.26	1.10–4.64	***0.026***
ITF																					
Tumor‐High	1				1			1				1									
Tumor‐Low	4.34	2.1–8.97	***<0.0001***		3.13	1.60–6.1	***0.00082***	3.96	1.82–8.60	***0.00052***		2.21	1.06–4.64	***0.035***							

Abbreviations: CI, confidence interval; DOI, depth of invasion; HR, hazard ratio; ITF, invasive tumor front; PNI, perineural invasion; POI, pattern of invasion; WPOI, worst POI.

*Statistically significant differences (*p* < 0.05) indicated in bold type.

In inner tumor, although only 25.44% (29/114) of patients were classified into the tumor‐low group (low TSR), patients had a poorer prognosis when the proportion of tumor area in the inner tumor was low. Our results also confirmed this conclusion: OS, DFS, MFS, and RFS were significantly decreased in patients in the tumor‐low group (low TSR) (Figure 3D–F, Figure S1B). The results of the Cox proportional hazards model showed that the TSR of the inner tumor was an independent prognostic factor for OS (HR = 2.46, 95% CI 1.18–5.13; *p* = 0.016), DFS (HR = 2.26, 95% CI 1.10–4.64; *p* = 0.026) and RFS (HR = 2.95, 95% CI 1.07–8.18; *p* = 0.037) (Table 2, Table S1).

### 3.3 Correlation of clinicopathological variables with the TSR

**FIGURE 4:**
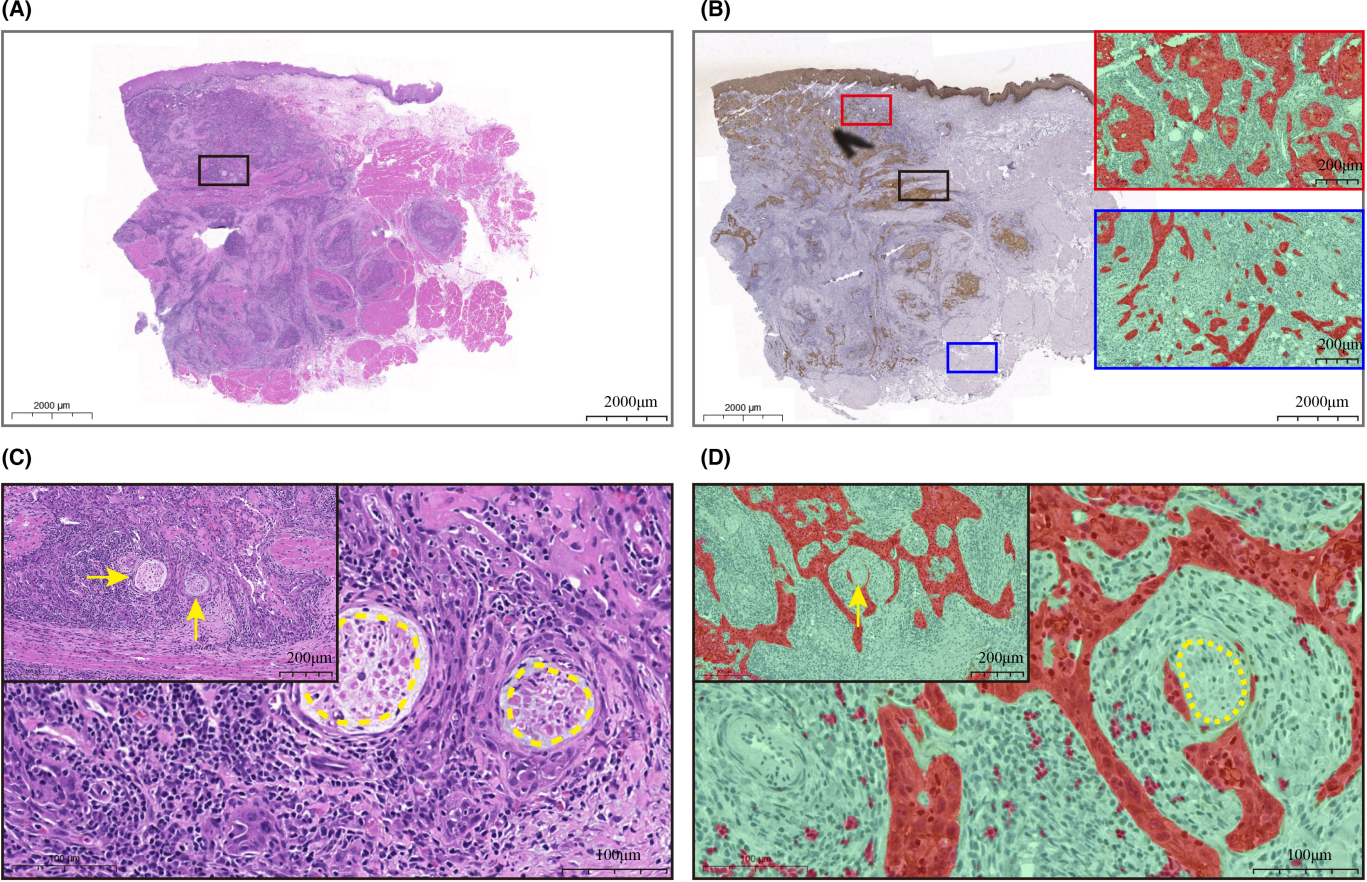
Schematic diagram of PNI^+^ in serial slides. (AB) HE (A) and CK (B) stained WSIs of serial slides; the black box in the middle position is the location where PNI occurred. (B) On the right are the overlay images after ImageJ segmentation recognition classification, with the inner tumor on top and the ITF on the bottom. (C) Representative image of PNI^+^ in an HE slide; the yellow arrow and dashed box are nerves, which are surrounded by tumor cells. D: Overlay image of a CK‐stained slide after ImageJ segmentation recognition classification; the yellow dashed line is the nerve, which is surrounded by tumor cells.

To assess the specific relationship between clinicopathological variables that were correlated with the TSR distribution in the chi‐square analysis and the proportion of tumor area (Table 1), we performed additional analyses. Correlation analysis was performed to compare the proportion of tumor area within the T‐stage, N‐stage, POI, WPOI, DOI, and PNI groups. The results showed that there was a significant relationship between the N stage and the proportion of tumor area for both the inner tumor and the ITF (*p* values of 0.035 and 0.028, respectively) (Figure S2C,D), which was the same as the results of previous works.^8^ In the N2 group, the percentage of patients with a low TSR in the ITF was as high as 62.96% (Figure S2D). The POI also had a high correlation with the proportion of tumor area (*p* values of 0.021 and 0.0019) (Figure S2E,F). However, among the WPOIs, only the WPOI of the ITF correlated with the proportion of tumor area (*p* = 0.0045) (Figure S2G,H). Furthermore, we found a strong correlation between the PNI and the proportion of tumor area (Figure S2K,L, Figure 4), suggesting that we might be able to predict the PNI with the TSR.

Table S2 shows the association between PNI and other clinicopathological characteristics. Regarding predictors of PNI+, univariate logistic regression analysis revealed histological parameters significantly associated with PNI+ risk, including POI (*p* < 0.0001), WPOI (*p* = 0.011), DOI (*p* = 0.0019), inner tumor‐TSR (*p* = 0.00019), and ITF‐TSR (*p* = 0.00049). In multivariate logistic regression analysis, inner tumor‐TSR (*p* = 0.014) and DOI (*p* = 0.0027) remained significant predictors of PNI + after controlling for the above five factors.

## 4 DISCUSSION

In this study, we quantified the TSR in different histological zones in OSCC using CK‐stained WSI through the TWS plug‐in of the open‐source software ImageJ. We further demonstrated that the TSR based on semi‐automated software analysis of inner tumor was an independent prognostic factor for OS, DFS, MFS, and RFS in OSCC patients and TSR for ITF was an independent prognostic factor for OS, DFS and MFS in OSCC patients. We also found that the TSR of the inner tumor and DOI independently predicted PNI^+^ status in OSCC.

Our study confirms the results of previous investigations; that is, the proportion of tumor area in tumors is an independent factor in the prognosis of OSCC patients. This maintains the consistency of these findings with those of several studies of head and neck cancers.^3^, ^5^, ^8^, ^14^, ^22^, ^23^, ^24^, ^25^, ^26^, ^27^ In a study of early‐stage oral tongue squamous cell carcinoma (OTSCC), stroma high was found to be an independent risk factor for tumor recurrence and cancer‐related mortality.^25^ Another study of OTSCC found no significant difference in TSR between older and younger patients, which may indicate a good consistency of TSR among patients of different ages.^28^ Several other studies of oral cancer have similarly shown that stroma‐high predicts a poorer prognosis for patients.^14^, ^22^, ^23^, ^29^ In addition, it has also been shown that TSR of biopsy samples has 86.8% agreement with TSR of postoperative tissue sections, which could broaden the application of TSR.^30^ We further hypothesize that the TSR of biopsy samples has a higher consistency with the TSR of the inner tumor of postoperative tissue sections, which we hope to confirm in future studies.

However, the analytical methods used in these studies are directly quoted from those for colon cancer,^31^ and the image field used in TSR analysis is located in the ITF or the area with highest amount of stroma. The one‐sidedness of such studies ignores the role of inner tumor in prognosis prediction for OSCC patients. Our results showed that the TSR was generally higher in inner tumor than in the ITF, but it was an independent prognostic factor for OS, DFS, and RFS in OSCC patients, that is when the proportion of tumor area in the inner tumor was low, patients had a worse prognosis. In addition, we found that TSR of the inner tumor was an independent prognostic factor for RFS, but ITF was not. One possible reason for this is that when ITF stroma proportion is high, the stroma proportion of the inner tumor is present in two situations (Figure 2B,C). In this case, the overall malignant phenotype and motility of the tumor is relatively neutralized and may be poorer in predicting recurrence. To our knowledge, this is the first study to partition pathological images and quantify TSR analysis for both inner tumor and ITFs; inner tumor also shows good prognostic prediction performance for OSCC patients, which can play an important role when ITFs cannot be determined from tissue slides (tumors of the gingiva or hard palate, tumor reduction surgery samples, biopsy samples, and samples with ITF lost due to irregular sampling). It can also greatly accelerate the clinical diagnosis process of TSR.

We also evaluated the relationship between multiple clinical parameters and the TSR. The T stage did not correlate with the proportion of tumor cell area (*p* values of 0.19 and 0.22 for inner tumor and ITF, respectively) (Figure S2A,B). This is different from the results of Karpathiou et al.,^24^ and we speculate that in addition to the difference in anatomical subunits, it may also be due to the fact that TSR is lower in both inner tumors and ITF when tumor cells have strong dissociative and motility abilities. The proportion of tumor cells in this case does not correlate with the size of the tumor. The N stage, on the other hand, showed an increase in the number of patients with a low proportion of tumor area with increasing stage, as expected and consistent with the results of previous studies.^8^ In the POI and WPOI, this trend also follows our speculation that the number of patients with a low proportion of tumor area increases with increasing invasion level, which is related to the high overlap between the definitions of POI and WPOI and TSR.^10^ We did not find a significant association between DOI and TSR, which is different from the results of other studies.^22^, ^25^, ^32^ This may be related to the setting of the study cohort, the anatomical subunits. Subsequent prospective studies in large head and neck cancer cohorts should be considered to assess TSR and analyze the correlation between TSR and clinicopathological parameters.^4^ PNI, on the other hand, demonstrated a high correlation with TSR that far exceeded other clinical parameters, which is consistent with the findings of Almangush et al.^25^ Previous studies have shown that tumor cells can migrate along nerves^33^, ^34^ and that tumor cells with low TSRs may possess a stronger ability to migrate. We have not found further studies on the association between PNI and the proportion of tumor area, and more research is needed to explain this phenomenon.

Our study has several limitations. First and most importantly, it was retrospective, and the sample size was relatively small. This resulted in our inability to analyze the survival of patients with similarly expressed TSRs in both the inner tumor and ITF. Another reason is that when the TSR of the inner tumor is low, the TSR of the ITF is often low. In addition, the small size of some of the tissue slides caused a certain degree of overlap in our selection of image fields; consequently, the distribution characteristics of tumor cells in the two histological regions were not adequately represented. We also did not provide a biological theoretical basis for the association between the TSR and PNI.

In conclusion, we found that the TSRs of the inner tumor and ITF can predict the prognosis of OSCC patients and that the TSR of the inner tumor can independently predict the occurrence of PNI.

## AUTHOR CONTRIBUTIONS

**Shuai Wang:** Conceptualization (lead); data curation (lead); formal analysis (equal); investigation (lead); methodology (lead); resources (equal); supervision (equal); validation (equal); visualization (equal); writing – original draft (equal); writing – review and editing (equal). **Qian Si:** Data curation (equal); formal analysis (equal); investigation (equal); validation (equal); writing – original draft (equal). **Yan Wu:** Conceptualization (equal); data curation (equal); formal analysis (equal); investigation (equal); software (equal). **Yawei Sun:** Data curation (equal); formal analysis (equal); software (equal). **Weixian Zhang:** Data curation (equal); software (equal). **Xiaofeng Huang:** Data curation (equal); supervision (equal). **Tao Zeng:** Formal analysis (equal); methodology (equal); software (equal). **Shen Chen:** Data curation (equal). **Xihu Yang:** Funding acquisition (equal); investigation (equal); methodology (equal); project administration (equal); supervision (equal); writing – review and editing (equal). **Yanhong Ni:** Funding acquisition (equal); investigation (equal); methodology (equal); project administration (equal); supervision (equal); writing – review and editing (equal). **Qingang Hu:** Funding acquisition (equal); investigation (equal); methodology (equal); project administration (equal); supervision (equal); writing – review and editing (equal).

## FUNDING INFORMATION

This research was funded by the National Natural Science Foundation of China (82173159, 81902759, 82002865, 82173380), the Key Research and Development Projects in Jiangsu Province (No. BE2020628), Doctoral Program for Entrepreneurship and Innovation of Jiangsu Province (JSSCBS20211596), and Nanjing Medical Science and Technique Development Foundation (Nos. YKK19091, YKK20151).

## CONFLICT OF INTEREST STATEMENT

The authors declare no potential conflicts of interest.

## ETHICS STATEMENT AND CONSENT TO PARTICIPATE

This study was approved by the Medical Ethics Committee of Nanjing Stomatology Hospital, Medical School of Nanjing University (NJSH‐2021NL‐025). As a retrospective study, this study did not require informed consent.

## Supporting information


Data S1.

## Data Availability

Raw data for this study were generated at department of oral pathology, Stomatological hospital, Medical School of Nanjing University. Derived data supporting the findings of this study are available from the corresponding author upon request.

## Footnotes

1 *Statistically significant differences (*p* < 0.05) indicated in bold type.
