# Supplementary material for: Multiperspective quantitative tumor–stroma ratio reveals histological areas associated with poor outcomes in oral squamous cell carcinoma
Source: Cancer Med. 2023 May 15;12(11):12161–72. doi: 10.1002/cam4.5909 (PMC10278530; doi:10.1002/cam4.5909)
Supplement: Supplementary file 1 — Data S1. [file CAM4-12-12161-s001.docx]

**Title:** Multiperspective quantitative tumor-stroma ratio reveals histological areas associated with poor outcomes in oral squamous cell carcinoma

**Running title:** TSR Analysis in different regions of OSCC

**Authors:** Shuai Wang^1,2#^, Qian Si^1,3#^, Yan Wu^1,3^, Yawei Sun^2^, Weixian Zhang^1,2^, Xiaofeng Huang^3^, Tao Zeng^4^, Sheng Chen^3^, Xihu Yang^5*^, Yanhong Ni^1*^, Qingang Hu^1,2*^

**Affiliations:**

^1^Central Laboratory of Stomatology, Nanjing Stomatological Hospital, Medical School of Nanjing University, Nanjing, 210008, Jiangsu, China.

^2^Department of Oral and Maxillofacial Surgery, Nanjing Stomatological Hospital, Medical School of Nanjing University, Nanjing, 210008, Jiangsu, China.

^3^Department of Oral Pathology, Nanjing Stomatological Hospital, Medical School of Nanjing University, Nanjing, 210008, Jiangsu, China.

^4^State Key Lab of Pharmaceutical Biotechnology, College of Life Sciences, Nanjing University, Nanjing, 210008, Jiangsu, China.

^5^Department of Oral and Maxillofacial Surgery, Affiliated Hospital of Jiangsu University, Zhenjiang, 212001, Jiangsu, China.

**The E-mail Address of Each Author:**

Shuai Wang: [shuaiwang@smail.nju.edu.cn](mailto:shuaiwang@smail.nju.edu.cn); Qian Si: [sq_oral@foxmail.com](mailto:sq_oral@foxmail.com); Yan Wu: [1711499302@qq.com](mailto:1711499302@qq.com); Yawei Sun: [sunyaweinju@163.com](mailto:sunyaweinju@163.com); Weixian Zhang: [zhangweixian2020@163.com](mailto:zhangweixian2020@163.com); Xiaofeng Huang: hxf681008@sina.com; Sheng Chen: [jakjone@163.com](mailto:jakjone@163.com); Tao Zeng: [1940375404@qq.com](mailto:1940375404@qq.com); Xihu Yang: [yangxihu1981@126.com](mailto:yangxihu1981@126.com); Yanhong Ni: [yanhong.ni@nju.edu.cn](mailto:yanhong.ni@nju.edu.cn); Qingang Hu: qghu@nju.edu.cn.

#Co-first authors contributed equally to this article.

* Co-corresponding authors:

Xihu Yang, Department of Oral and Maxillofacial Surgery, Affiliated Hospital of Jiangsu University, Zhenjiang, 212001, Jiangsu, China. Email: yangxihu1981@126.com

Yanhong Ni, Central Laboratory of Stomatology, Nanjing Stomatological Hospital, Medical School of Nanjing University, Nanjing, Jiangsu, 210008, China. Email: yanhong.ni@nju.edu.cn.

Qingang Hu, Department of Oral and Maxillofacial Surgery, Nanjing Stomatological Hospital, Medical School of Nanjing University, Nanjing, Jiangsu, 210008, China. Email: qghu@nju.edu.cn

**Supplementary Figure S1**


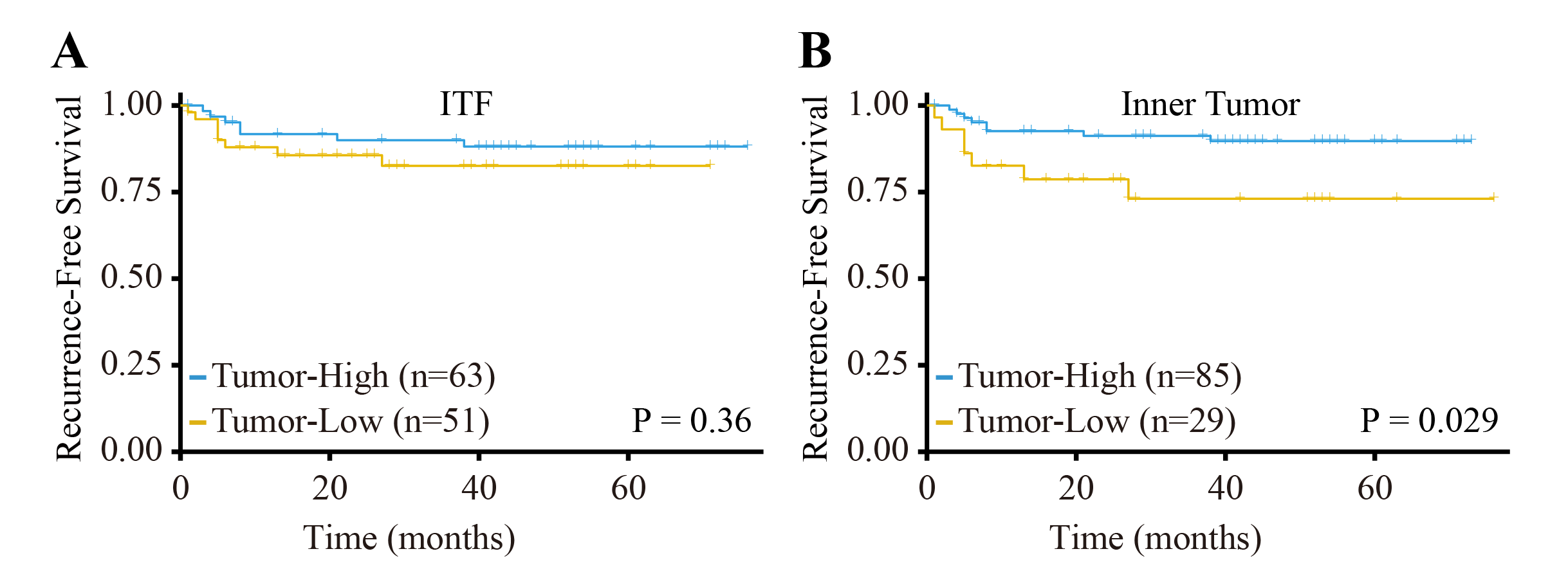


**Supplementary Figure S1:** Kaplan‒Meier curve of the TSR. A: There was no significant association between RFS and TSR distribution in the ITF. B: Patients with OSCC had a poorer prognosis when the proportion of tumor area in the inner tumor is low.

**Supplementary Figure S2**


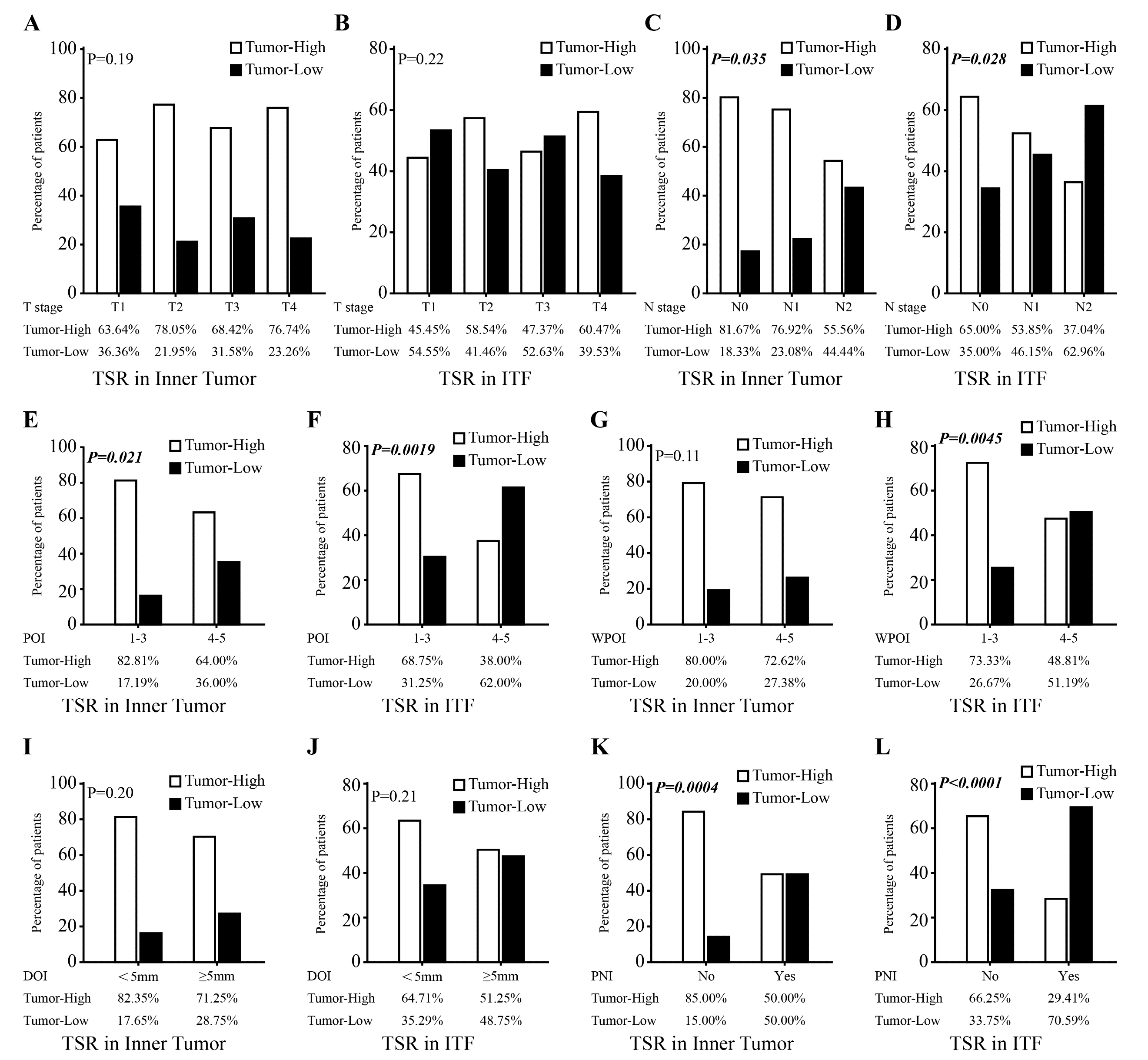


**Supplementary Figure S2:** Correlation of the TSR with clinicopathological variables. Correlations of the T stage, N stage, POI, WPOI, DOI and PNI with the TSR of the inner tumor and ITF were analyzed.

**Supplementary Table S1:** Univariate and multivariate analyses including sex, age, clinical stage, differentiation, smoking, POI, WPOI, DOI, PNI and MFS and RFS of the two types of TSR.


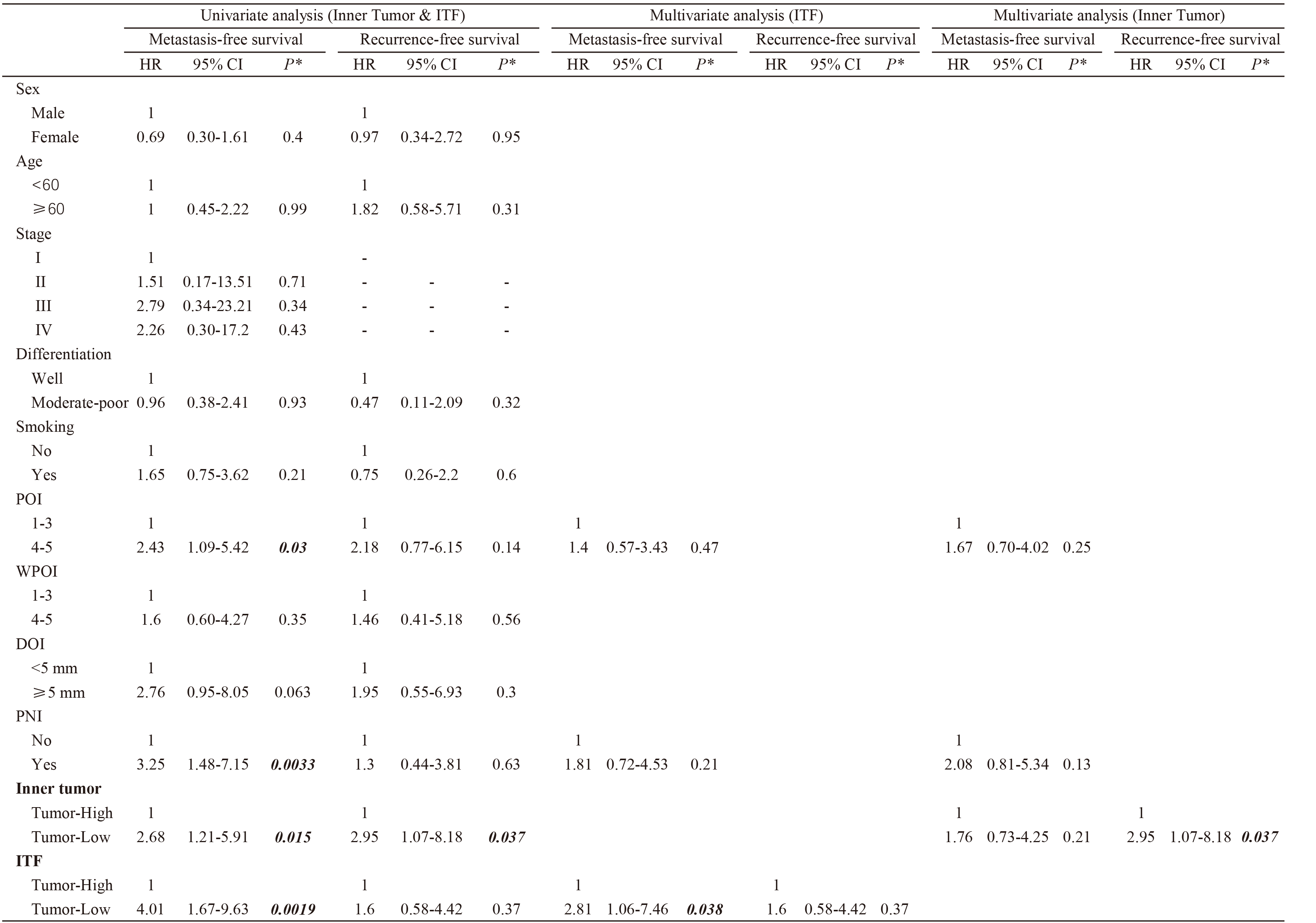


ITF: invasive tumor front; POI: pattern of invasion; WPOI: worst POI; DOI: depth of invasion; PNI: Perineural Invasion; OR: odds ratio; CI: confidence interval. *Statistically significant differences (p<0.05) indicated in bold type.

**Supplementary Table S2:** Univariate and multivariate logistic regression analyses of clinicopathological parameters predicting PNI+.


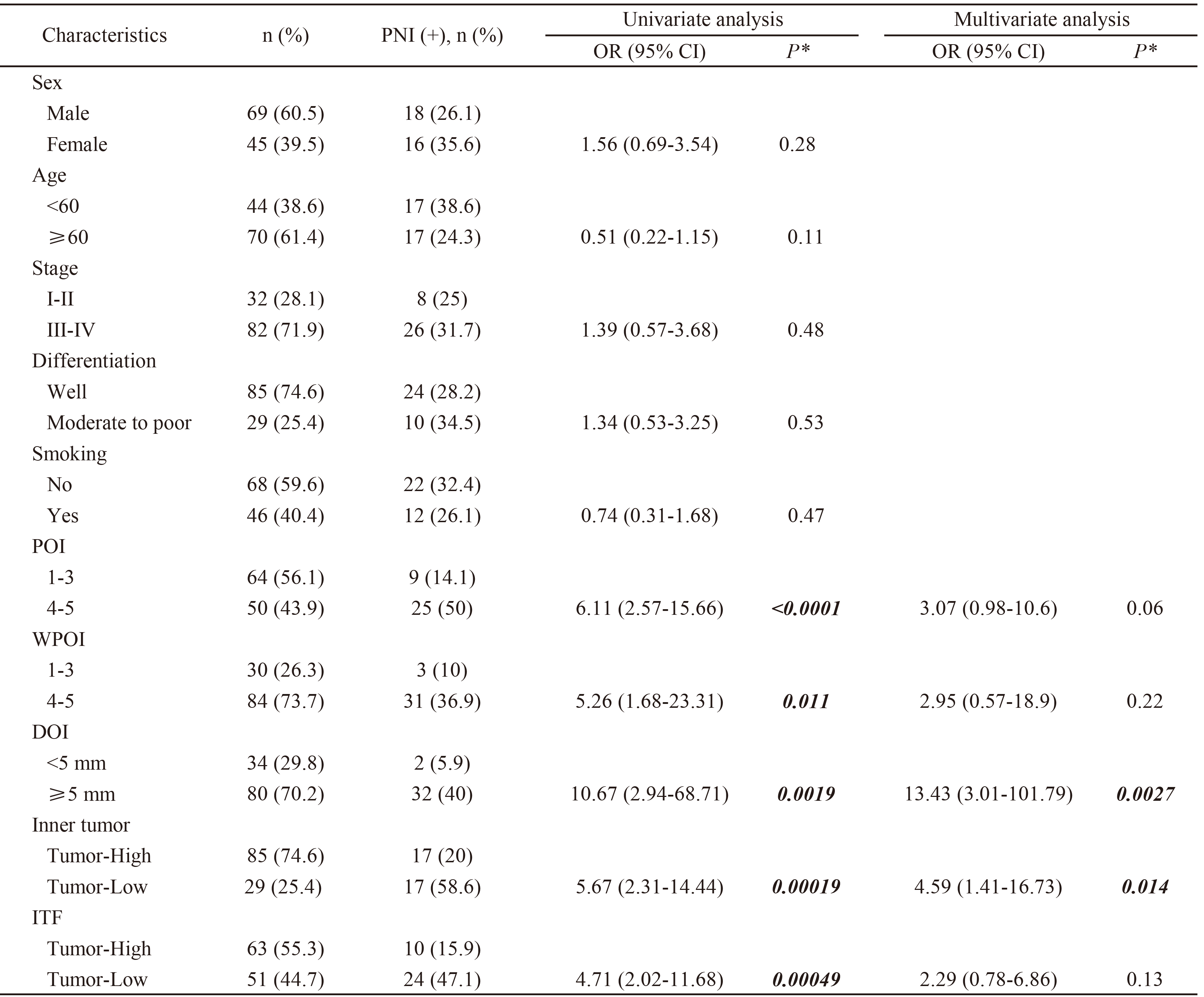


ITF: invasive tumor front; POI: pattern of invasion; WPOI: worst POI; DOI: depth of invasion; PNI: Perineural Invasion; OR: odds ratio; CI: confidence interval. *Statistically significant differences (p<0.05) indicated in bold type.
